# Potential Diagnostic and Prognostic Values of CBX8 Expression in Liver Hepatocellular Carcinoma, Kidney Renal Clear Cell Carcinoma, and Ovarian Cancer: A Study Based on TCGA Data Mining

**DOI:** 10.1155/2022/1372879

**Published:** 2022-06-29

**Authors:** Jie Lin, Lizhu Chen, Dingjie Wu, Jiexiang Lin, Bin Liu, Ciren Guo

**Affiliations:** ^1^Department of Gynecology, Fujian Medical University Cancer Hospital, Fujian Cancer Hospital, Jinan District, Fuzhou, Fujian Province, China; ^2^Department of Abdominal Oncology, Fujian Medical University Cancer Hospital, Fujian Cancer Hospital, Jinan District, Fuzhou, Fujian Province, China; ^3^Department of Microbial and Biochemical Pharmacy, School of Pharmacy, China Medical University, Shenyang, Liaoning Province, China; ^4^Shengli Clinical Medical College, Fujian Medical University, Fuzhou, China

## Abstract

**Background:**

Chromobox protein homolog 8 (CBX8), a transcriptional repressor, participates in many biological processes in various carcinomas. Cell differentiation, aging, and cell cycle progression are examples of such processes. It is critical to investigate CBX8 expression and its relationship with clinicopathological characteristics in liver hepatocellular carcinoma (LIHC), kidney renal clear cell carcinoma (KIRC), and ovarian cancer (OV) to investigate CBX8's potential diagnostic and prognostic values.

**Methods:**

TCGA and CPTAC databases were used to compare the data between cancer and matched normal tissues on RNA and protein expression profiles and their relevant clinical information to determine the relationship between CBX8 and clinicopathological features. Kaplan–Meier analyses were used to assess CBX8 relationship's with disease-free survival (DFS), relapse-free survival (RFS), and overall survival (OS). The multivariate Cox regression analysis was used to identify independent risk factors which affect prognosis. DNA methylation and genetic changes and their impact on prognoses were evaluated by cBioPortal and MethSurv websites. Spearman's correlation was used to determine the relationship of CBX8 expression with somatic mutation. Tumor immune estimation resource (TIMER) was adopted to investigate the relationship between CBX8 and immune cell infiltration (ICI). CBX8-relevant genes and proteins are analyzed by EnhancedVolcano and STRING databases. The gene set enrichment analysis (GSEA) was performed to investigate the potential functions of CBX8.

**Results:**

CBX8 RNA and protein overexpression were confirmed in LIHC, KIRC, and OV (*p* < 0.05). High CBX8 was significantly related to the clinical features and poor prognoses. The CBX8 genetic alteration rate was 3%. DNA methylation was also associated with prognoses. CBX8 closely interacted with ICI, TMB, MSI, purity, and ploidy. GO analyses revealed that CBX8-associated genes were enriched in biological processes, cell cycle regulation, and molecular functions. KEGG analyses exhibited that CBX8 was gathered in signaling pathway regulation. GSEA revealed that cell cycle, DNA replication, and Wnt signaling pathways were differentially enriched in the high CBX8 expression group.

**Conclusions:**

CBX8 could be a potential diagnostic and prognostic biomarker for LIHC, KIRC, and OV cancers.

## 1. Introduction

Chromobox protein homolog 8 (CBX8), regarded as human polycomb 3, is the core member of CBX family [1]. CBX proteins are involved in many biological courses, like pluripotency maintenance and self-renewal in developmental program controls, cell fate decisions, and embryonic stem cells. CBX8 regulates cell differentiation, aging, and cell cycle progression in many malignant tumors [2–4]. Evidence implies that CBX8 expression is closely related to tumor generation and growth, but CBX8's role in liver hepatocellular carcinoma (LIHC), kidney renal clear cell carcinoma (KIRC), and ovarian cancer (OV) and its link with prognoses and clinicopathological characteristics of patients remain elusive. In this study, relative clinical information and a significant expression profile data of patients of LIHC, KIRC, and OV were retrieved from TCGA [5, 6] for estimating the clinical values of CBX8.

## 2. Methods

### 2.1. Comparison of the CBX8 Expression Level

Relative clinical information and RNA expression profiles of LIHC, KIRC, and OV patients were downloaded from TCGA database (https://portal. http://gdc.cancer.gov/), and the information of ovaries were obtained from GTEx database (https://commonfund.nih.gov/GTEx/). The exclusion criteria were as follows: (1) the loss of CBX8 expression; (2) follow-up information absence in survival analysis; (3) uncertain TNM phase; and (4) accompaniment of other tumors. Finally, 374 LIHC tumor tissue vs. 50 normal liver tissue, 539 KIRC tumor tissue vs. 72 normal kidney tissue, and 379 OV tumor tissue vs. 180 normal ovary tissue were included in our study. Protein expression profiles were obtained through CPTAC database (https://cptac-data-portal.georgetown.edu/datasets), and Human Protein Atlas database (https://www.proteinatlas.org) was employed to identify tumor-type-specific protein expression patterns.

### 2.2. Correlation Analysis of CBX8 with Clinicopathological Characteristics and Prognoses

LIHC, KIRC, and OV patients are divided into low and high CBX8 expression groups when adopting optimal CBX8 mRNA expression as the cutoff value based on R (version 4.0.5). Correlation of CBX8 and clinicopathological characteristics such as age, gender, stage of cancer, and histological grade were analyzed by utilizing the chi-square test package of R. The prognosis of patients (OS, RFS, and DFS) was evaluated by Kaplan–Meier plots.

### 2.3. Somatic Mutation Analysis

Somatic mutations were visualized using cBioPortal (http://cbioportal.org/). Change frequencies of CBX8 in LIHC, KIRC, and OV patients were analyzed. Genomic mutations of CBX8 contained missense mutation, deep deletion, and copy number amplification. Kaplan–Meier plots were created to identify difference significance between survival plots, and *p* < 0.05 was statically evident.

### 2.4. DNA Methylation Analysis

The MethSurv database (https://biit.cs.ut.ee/methsurv/) analyzed CBX8 gene DNA methylation sites of LIHC and KIRC. The DNA methylation data of OV was downloaded from TCGA database. Moreover, the overall survival of cg07581365 methylation was evaluated among LIHC, KIRC, and OV.

### 2.5. Analyses of Associations between CBX8 Expression and TMB, Microsatellite Instability (MSI), Purity, and Ploidy

Tumor mutation burden (TMB) scores were computed by R (version 4.0.5) [7]. The microsatellite instability (MSI), purity, and ploidy of these three cancers were obtained from the Sangerbox website (http://vip.sangerbox.com/login.html). Through the Spearman correlation test, we assessed the associations of CBX8 expression with TMB, MSI, purity, and ploidy in LIHC, KIRC, and OV.

### 2.6. CBX8 Expression and ICI

Tumor immune estimation resource (TIMER) (http://cistrome.shinyapps.io/timer) [8] was adopted to infer the immune cell infiltration (ICI) and its relations with CBX8 in LIHC, KIRC, and OV.

### 2.7. Analysis of CBX8-Related Partners

We tried to screen out genes related to CBX8 expression and its targeting proteins to obtain more accurate knowledge of CBX8 molecular mechanisms in tumorigenesis. For RNA level, LIHC, KIRC, and OV patients were separately divided into low- and high-expression groups based on CBX8 medium expression as the cutoff value based on R package, and then EnhancedVolcano (version 1.11.3) was applied. After that, the STRING database was utilized for protein level to obtain PPI network information of CBX8 protein using the Cytoscape 3.5.0 instrument. Based on the integrated score, EED, BMI1, and RNF2 proteins were considered closely interacting with CBX8 protein. Then, Pearson's correlation analyses were conducted. Lastly, we divide LIHC patients into four groups based on mRNA expression (LL: low CBX8 and low gene A; LH: low CBX8 and high gene A; HL: high CBX8 and low gene A; and HH: high CBX8 and high gene A). Then, we created Kaplan–Meier plots and performed a log-rank test to identify the survival curves of different combination groups.

### 2.8. Gene Set Enrichment Analyses (GSEA)

Gene Ontology (GO) was applied to find the potential biological functions of CBX8 by adopting the clusterProfiler package [9]. Kyoto Encyclopedia of Genes and Genomes (KEGG) was curated from the Molecular Signature Database to feature apparent enrichment [10, 11]. By adopting the upper-tenth and lower-tenth values of CBX8 expressions of LIHC, KIRC, and OV patients, respectively, we performed GSEA (version 1.52.1) to decipher survival differences between high and low CBX8 expression groups [12].

### 2.9. Statistical Analyses

R (version 4.0.5) was used for statistical analysis. Comparisons between tumor and nontumor tissues were conducted by the two-sided Wilcoxon test. The chi-square test or Fisher's exact probability method was used to evaluate the correlation between CBX8 expression and clinical characteristics of patients. The Kaplan–Meier method was applied to estimate patients' OS, RFS, and DFS. Univariate and multivariate Cox regression analyses were performed to identify independent prognostic factors [13]. Clinicopathological parameters with *p* < 0.2 in the univariate analysis were incorporated into the multivariate analysis to identify independent prognostic factors for patients. *p* < 0.05 was considered statistically significant. The Spearman correlation test was utilized to evaluate the relationships between CBX8 expression and immune cell infiltrations (ICIs).

## 3. Results

### 3.1. mRNA and Protein Expression Patterns of CBX8

**Figure 1:**
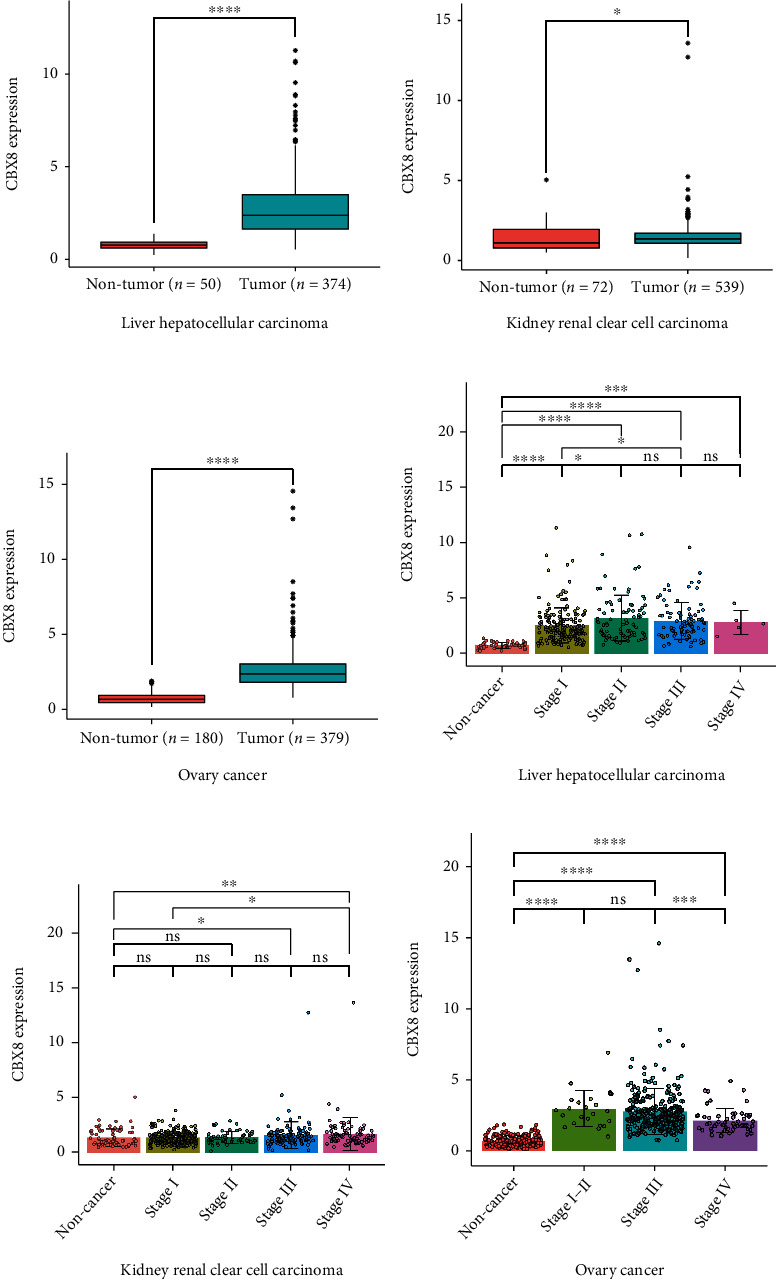
*CBX8* mRNA expression in three types of cancers. (a–c) *CBX8* mRNA expression in LIHC, KIRC, and OV tissues; (d–f) *CBX8* mRNA expression in different clinical stages of tumors; ns: nonsignificant; ^∗^*p* < 0.05, ^∗∗^*p* < 0.01, ^∗∗∗^*p* < 0.001, ^∗∗∗∗^*p* < 0.0001. Colored images are available online.

**Figure 2:**
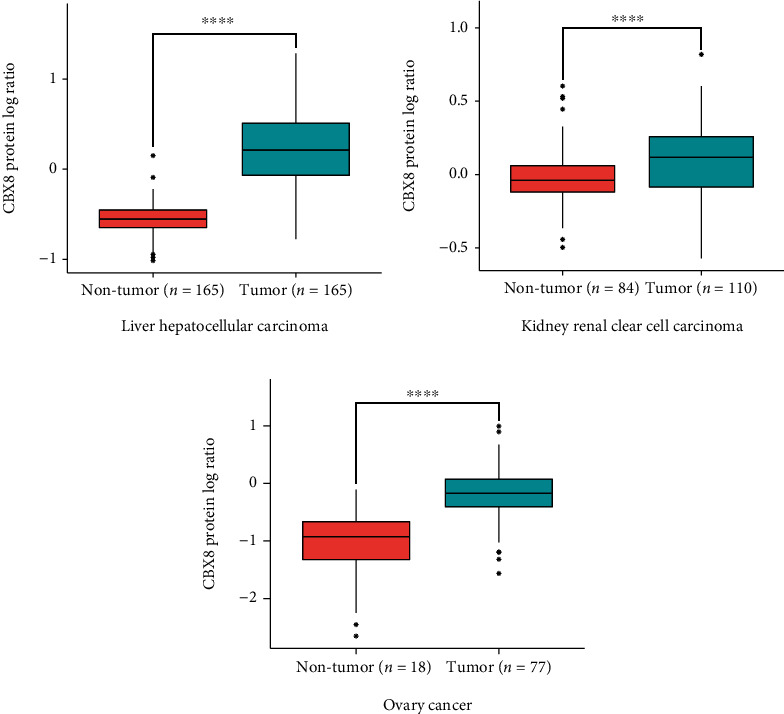
CBX8 protein expression in three types of cancers. (a–c) CBX8 protein expression in LIHC, KIRC, and OV tissues. ^∗^*p* < 0.05, ^∗∗^*p* < 0.01, ^∗∗∗^*p* < 0.001, ^∗∗∗∗^*p* < 0.0001.

**Figure 3:**
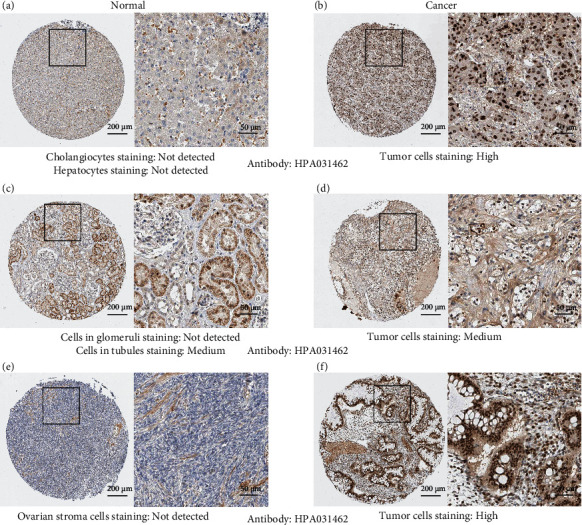
IHC analysis of CBX8 in three types of cancer. (a, b) CBX8 protein expression in normal tissue and LIHC tissue. (c, d) CBX8 protein expression in normal tissue and KIRC tissue. (e, f) CBX8 protein expression in normal tissue and OV tissue. IHC: immunohistochemistry. Colored images are available online.

CBX8 expressions in LIHC, KIRC, and OV tumor tissues were higher than in nontumor tissues (all *p* < 0.05, Figures 1(a)–1(c)). Subgroup analyses exhibited that CBX8 expression in nontumor groups was lower compared to tumor tissues of LIHC and OV patients in phases I-II (*p* < 0.05). However, in KIRC patients, CBX8 expression showed no differences between nontumor tissues and patients at stages I-II (*p* > 0.05). In all three cancers, CBX8 expression was significantly higher in cancer patients in III-IV phase compared to nontumor tissues (Figures 1(d)–1(f), *p* < 0.05). As for protein level, through the CPTAC database, higher CBX8 protein expression was observed in LIHC, KIRC, and OV tumor tissues compared to normal tissues (*p* < 0.05, Figure 2). Based on the Human Protein Atlas database, immunohistochemical staining of clinical specimens also identified the CBX8 level in LIHC, and OV tumor tissues exceeded compared to adjacent normal tissues (Figures 3(a), 3(b), 3(e), and 3(f)). For KIRC patients, CBX8 protein overexpression was found in tumor tissues compared with the glomeruli of normal kidney tissues. However, it showed no differences compared to the tubules of a normal kidney (Figures 3(c) and 3(d)).

### 3.2. CBX8 Expression Correlated with Clinicopathological Characteristics and Prognoses

**Figure 4:**
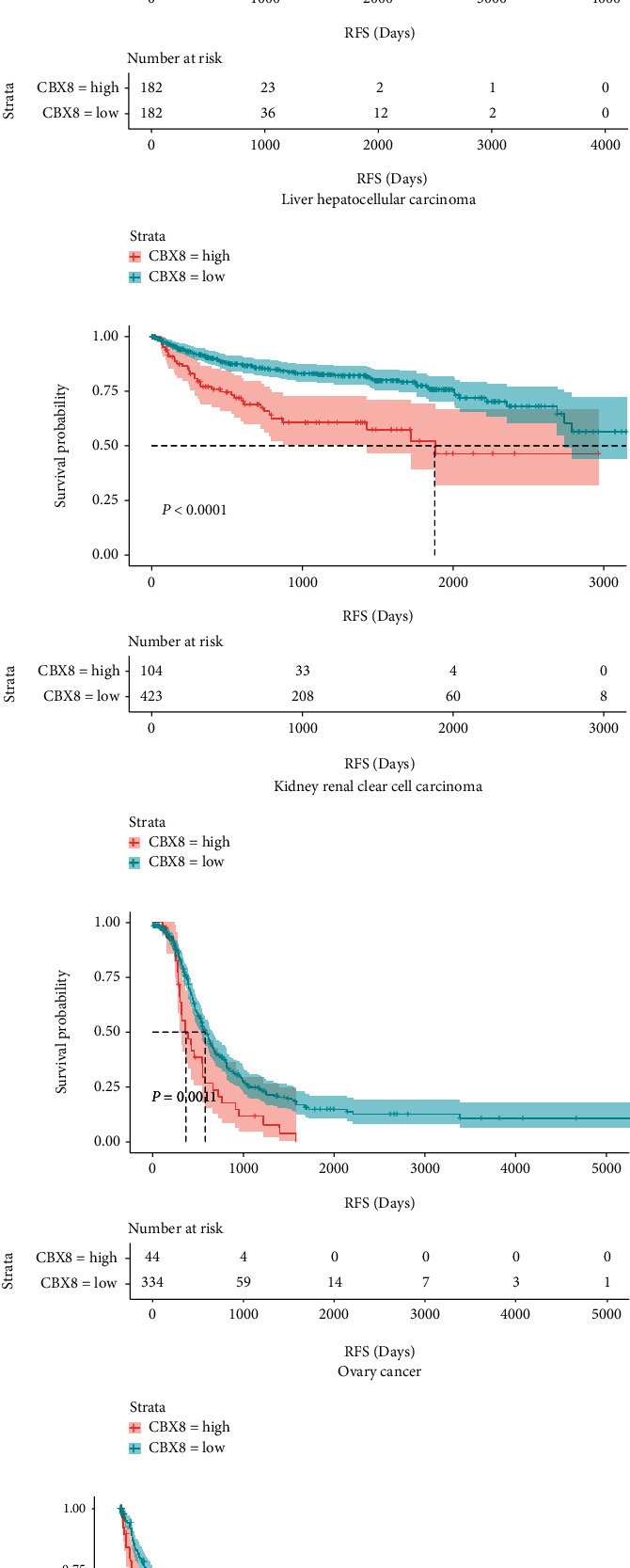
Survival analysis (harboring OS, RFS, and DFS) in three types of cancers. (a, d, g) Survival analysis for LIHC patients with different CBX8 expressions. (b, e, h) Survival analysis for KIRC patients with different CBX8 expressions. (c, f, i) Survival analysis for OV patients with different CBX8 expressions. OS: overall survival; RFS: recurrence-free survival. DFS: disease-free survival. Colored images are available online.

Among LIHC and KIRC patients, higher *CBX8* mRNA expression was related to the histological grade of patients (*p* < 0.05), but not to gender, age, and TNM stages (*p* > 0.05, Tables 1 and 2). Among OV patients, higher CBX8 expression was related to TNM stages (*p* < 0.05), but not to age and grade (*p* > 0.05, Table 3). LIHC patients with higher CBX8 expression have shorter OS (*p* < 0.0001), RFS (*p* = 0.0052), and DFS (*p* < 0.0001) (Figures 4(a), 4(d), and 4(g)). Similarly, KIRC patients with higher *CBX8* expression have shorter OS (*p* < 0.0001), RFS (*p* < 0.0001), and DFS (*p* < 0.0001) (Figures 4(b), 4(e), and 4(h)). OV patients of higher *CBX8* expressions have shorter RFS (*p* = 0.0011) and DFS (*p* = 0.011), but no OS (*p* = 0.1) (Figures 4(c), 4(f), and 4(i)).

### 3.3. Value of CBX8 in Diagnoses and Prognoses

For LIHC patients, the univariate analysis (UA) showed that *CBX8* expression and TNM were closely related to poor OS, RFS, and DFS (*p* < 0.05). Multivariate analyses (MA) implied that CBX8 expression and TNM were independent prognostic considerations (IPFs) for OS and DFS (*p* < 0.05), while TNM was IPF for RFS (*p* < 0.05, Table 4). For KIRC patients, UA exhibited that *CBX8* expression, age, TNM phase, and histological grade were IPFs for OS and DFS (*p* < 0.05). MA implied that *CBX8* expression, TNM, and histological grade were IPFs for OS (*p* < 0.05), and *CBX8* expression, TNM stage, age, and histological grade were IPFs for DFS (*p* < 0.05). UA showed that *CBX8* expression, TNM stage, and histological grade were IPFs for RFS (*p* < 0.05). MA implied that *CBX8* expression, TNM, and histological grade were IPFs for RFS (*p* < 0.05, Table 5). Among OC patients, UA presented that *CBX8* expression was linked to poor RFS and DFS (*p* < 0.05), and MA indicated that *CBX8* expression was IPF for RFS and DFS (*p* < 0.05, Table 6).

### 3.4. CBX8 Genetic Alteration in Patients with LIHC, KIRC, and OV

**Figure 5:**
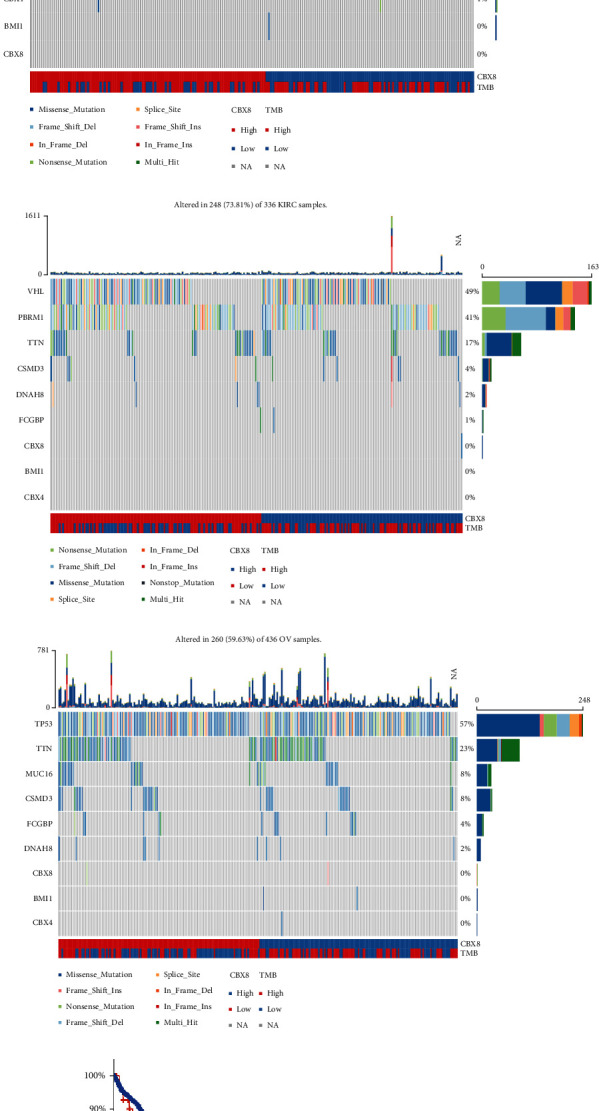
Analysis of genetic alteration and somatic mutation in CBX8 in LIHC, KIRC and OV. (a) OncoPrint visual summary of alteration on a query of CBX8. (b) Summary of alterations in CBX8 in LIHC, KIRC, and OV from TCGA. (c) Altercation frequency of CBX8 of LIHC, KIR, and OV cancers. (d) The somatic mutation rate of CBX8 across LIHC. (e) The somatic mutation rate of CBX8 across KIRC. (f) The somatic mutation rate of CBX8 across OV. Kaplan–Meier plots comparing (g) OS and (h) disease-free survival in patients with/without CBX8 gene alterations.

The somatic mutations of CBX8 gene in LIHC, KIRC, and OV were analyzed. The somatic mutation rate was 3% in three cancer types (Figure 5(a)). The most frequently mutated genes related to CBX8 somatic mutation in these three cancers are displayed in Figure 5(b), such as cbx4, enpp7, and cbx2. In LIHC, KIRC, and OV cancers, CBX8 amplification indicated relatively high change frequency, contributing to upregulating CBX8 expression (Figure 5(c)). Genetic mutations occurred among 211/364 LIHC samples (57.97%, Figure 5(d)). tp53 (28%), ttn (25%), muc16 (16%), and csmd3 (8%) were the most frequently mutated genes across LIHC cancer. Furthermore, missense mutation was the main mutation form. Among 336 KIRC cancer samples, genetic mutations occurred in 248 (73.81%, Figure 5(e)). vhl (49%), pbrm1 (41%), and ttn (17%) were the most frequently mutated genes across KIRC cancer. Of 436 OV cancer specimens, genetic mutations occurred in 260 (59.63%, Figure 5(f)). tp53 (57%), ttn (23%), muc16 (8%), and csmd3 (8%) were the most frequently mutated genes across OV cancer. Nonetheless, no obvious differences in genetic mutations were investigated across LIHC, KIRC, and OV cancer. Kaplan–Meier plots implied nonsignificant differences in OS (*p* = 0.109, Figure 5(g)) and DFS (*p* = 0.0878, Figure 5(h)) between patients featuring changes and those without changes across LIHC, KIRC, and OV cancers.

### 3.5. CBX8 Methylation in Patients with LIHC, KIRC, and OV

**Figure 6:**
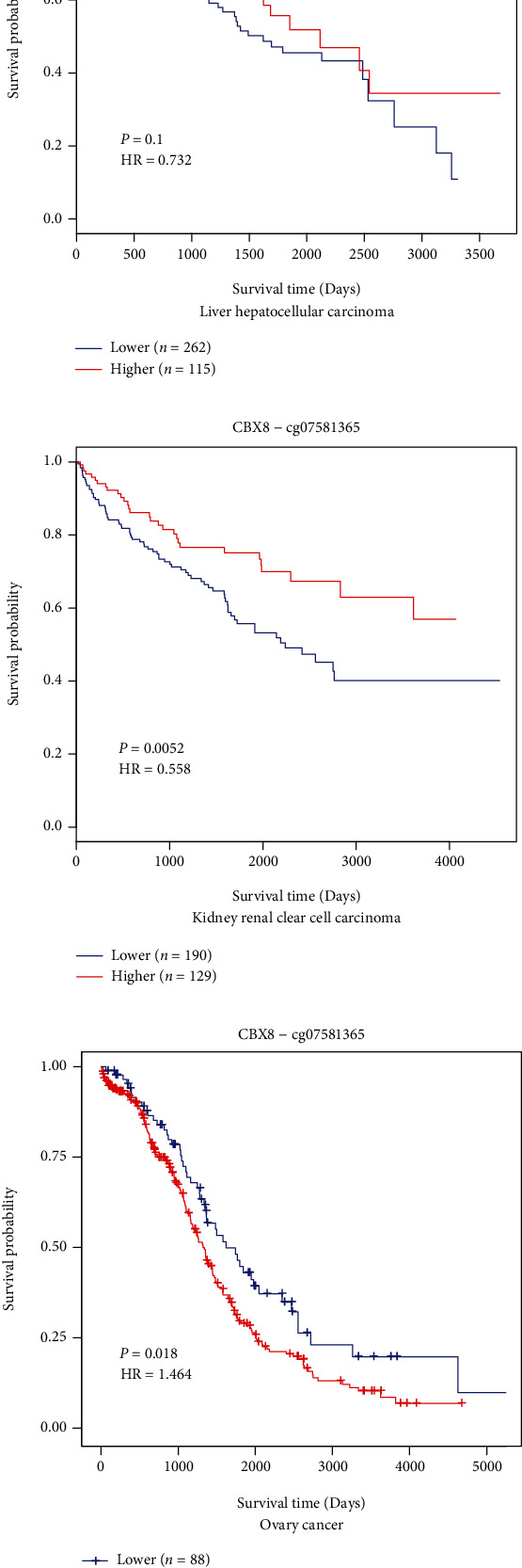
Survival analysis in three types of cancer based on the methylation levels of CBX8. (a) Survival analysis in LIHC patients with different methylation levels of CBX8. (b) Survival analysis in KIRC patients with different methylation levels of CBX8. (c) Survival analysis in OV patients with different methylation levels of CBX8. cg07581365 indicated the probe for methylation of CBX8. HR: hazard ratio. Colored images are available online.

DNA methylation levels of CBX8 in LIHC, KIRC, and OV with the prognostic value of the CpG site (cg07581365) were researched by adopting the MethSurv instrument. Outcomes implied that the CpG site methylation level (cg07581365) was correlated with prognosis. KIRC patients with higher CBX8 methylation exhibited better prognoses (*p* < 0.05, Figure 6(b)) and LIHC patients had the same trend (*p* > 0.05, Figure 6(a)). However, OV patients showed opposite results (*p* < 0.05, Figure 6(c)).

### 3.6. Link between CBX8 and ICIs

**Figure 7:**
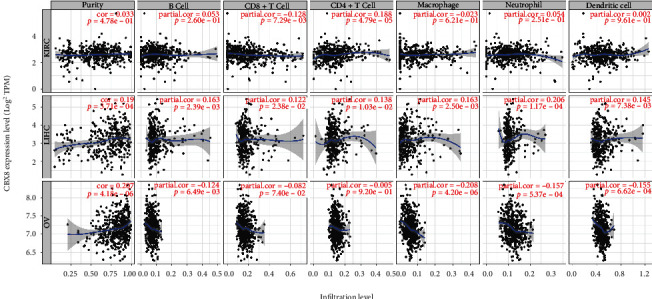
Relationship between CBX8 expression and immune cell infiltration in LIHC, KIRC, and OV.

The link between CBX8 expression and ICIs was adjusted by purity, B cells, CD8^+^ T cells, CD4^+^ T cells, macrophages, neutrophils, and DCs and was studied by TMER. In KIRC, the results proved that CBX8 expression was negatively related to infiltration of CD8^+^ T cells (*r* = −0.128, *p* = 7.29*e* − 03) and positively related to level CD4^+^ T cells (*r* = 0.188, *p* = 4.79*e* − 05). In LIHC, CBX8 expression was positively related to the infiltration of purity (*r* = 0.19, *p* = 3.71*e* − 04), B cells (*r* = 0.163, *p* = 2.39*e* − 03), CD8^+^ T (*r* = 0.122, *p* = 2.38*e* − 02), CD4^+^ T cells (*r* = 0.138, *p* = 1.03*e* − 02), macrophages (*r* = 0.163, *p* = 2.50*e* − 03), neutrophils (*r* = 0.206, *p* = 1.17*e* − 4), and DCs (*r* = 0.145, *p* = 7.38*e* − 03). In OV, CBX8 expression was positively related to the infiltration of purity (*r* = 0.207, *p* = 4.18*e* − 06) and negatively correlated with B cells (*r* = −0.124, *p* = 9.49*e* − 03), CD8^+^ T cells (*r* = 0.082, *p* = 7.40*e* − 02), macrophages (*r* = −0.208, *p* = 4.20*e* − 06), neutrophils (*r* = −0.157, *p* = 5.37*e* − 4), and DCs (*r* = −0.155, *p* = 6.62*e* − 04) (Figure 7).

### 3.7. Link between CBX8 Expression and TMB, Microsatellite Instability (MSI), Purity, and Ploidy

**Figure 8:**
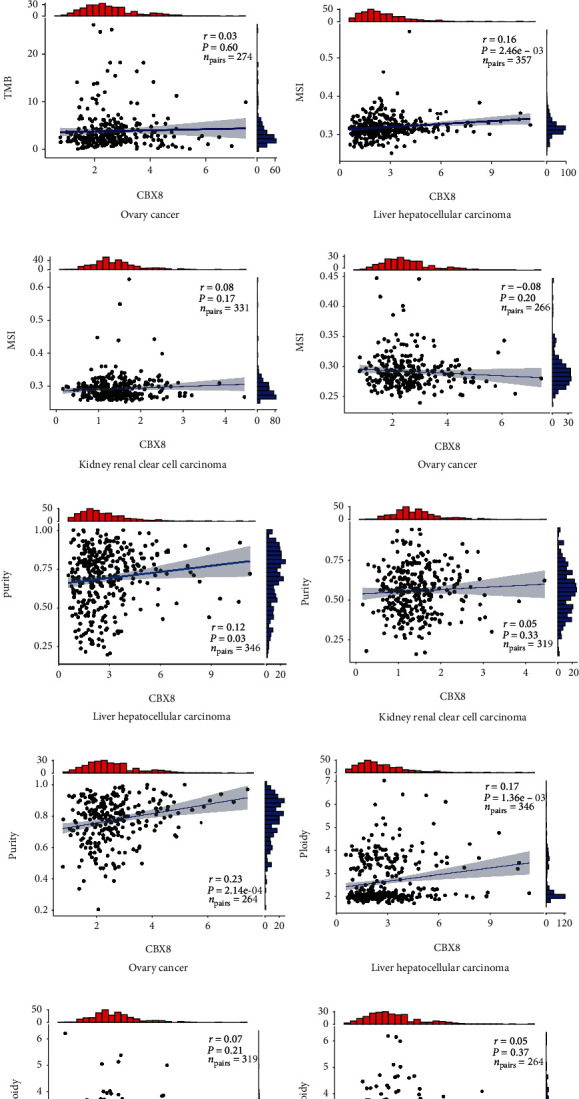
Correlation between CBX8 expression and biomarkers of immune cells in LIHC, KIRC, and OV cancers.

The investigation assessed the CBX8 expression correlation with TMB, MSI, and purity in LIHC, KIRC, and OV cancers. TMB, MSI, and purity serve as antitumor immunity and may predict therapeutic responses to immunotherapeutic agents. We also assessed the correlation between CBX8 expression and ploidy, linked to tumor heterogeneity. In Figures 8(a), 8(d), 8(g), and 8(j), CBX8 exhibited predominantly positive associations with the number of MIS, purity, and ploidy in LIHC (*p* < 0.05). No obvious correlation existed between CBX8 expression and TMB in LIHC. In KIRC, CBX8 exhibited predominantly positive associations with the number of TMB (*p* < 0.05) and showed no obvious correlation between MIS, purity, and ploidy (Figures 8(b), 8(e), 8(h), and 8(k)). In OV patients, CBX8 exhibited positive associations with the number of purity and showed no correlation between TMB, MIS, and ploidy (Figures 8(c), 8(f), 8(i), and 8(l)).

### 3.8. Identifying CBX8-Relevant Genes and Their Biological Function

The upregulation and downregulation of CBX8-related genes in high CBX8 expression samples of LIHC, KIRC, and OV were analyzed (Figure S1). We also showed the first 20 upregulated and downregulated CBX8-relevant genes ranked by ∣log2foldchange∣ among LIHC, KIRC, and OV (Tables S1-3). Notably, LIHC, KIRC, and OV cancers had the 40 same upregulated genes (Figure S2), such as FOXJ1, LOC100128674, MAGEC1, and PAGE2 (Table S4).

**Figure 9:**
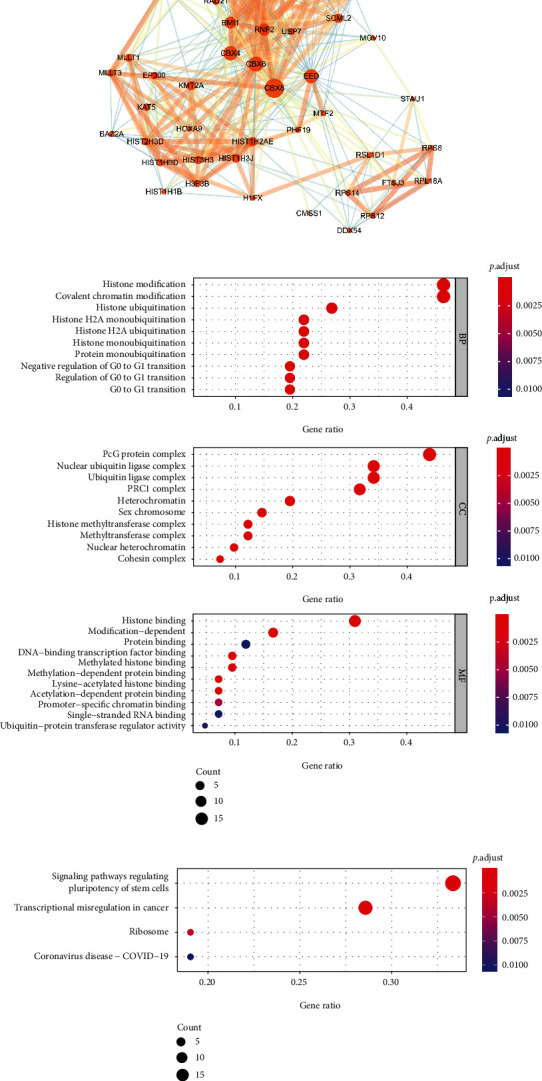
Gene enrichment analysis for CBX8-related genes. (a) PPI networks of CBX8-related proteins. The increase from a small circle to a bigger circle represents an increase in the number of genes interacting directly with each other (namely degree). (b) GO analysis; (c) KEGG analysis. PPI: protein-protein networks; GO: Gene Ontology; KEGG: Kyoto Encyclopedia of Genes and Genomes pathway. Colored images are available online.

**Figure 10:**
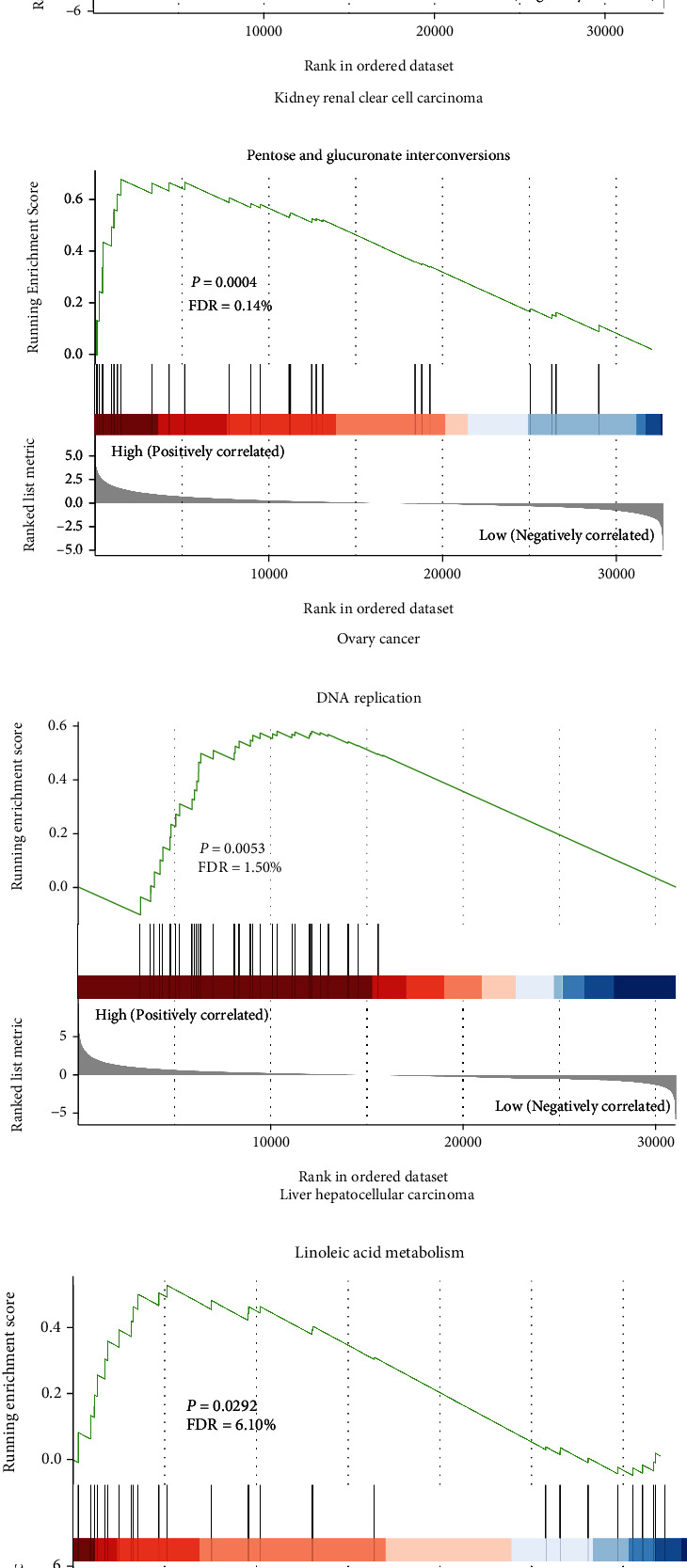
(a–f) GSEA was performed to explore the biological functions of high CBX8 expression in LIHC, KIRC, and OV.

A CBX8 protein PPI network of 50 proteins was constructed based on the STRING database, such related proteins as EED, BMI1, and RNF2 (Figure 9(a)). In LIHC, Pearson's correlation analyses implied that CBX8 was positively related to EED, BMI1, and RNF2 (Figures S3A-C; *p* < 0.001). In all gene combinations, patients in LL group had the longest OS (median survival time: 2001, 2080, and 1967 days, respectively). In the CBX8-EED group, LL patients had the longest OS (*p* = 0.00085), RFS (*p* = 0.015), and DFS (*p* = 0.00052) (Figures S3D, G, and J). The same result was also found in CBX8-BMI1 and RNF2 groups (CBX8-BMI1 OS (*p* = 0.00085), RFS (*p* = 0.015), and DFS (*p* = 0.00052) (Figures S3E, H, and K)) and RNF2 group OS (*p* = 0.00085), RFS (*p* = 0.015), and DFS (*p* = 0.00052) (Figures S3F, I, and L). GO analyses implied that CBX8-associated genes majorly enriched three main biological functions, biological process, regulation of cell cycle, and molecular functions (Figure 9(b)). KEGG analyses revealed that most CBX8-associated genes were enriched in transcriptional misregulation in cancer, signaling paths, regulating pluripotency of stem cells, systemic lupus, erythematosus, and other processes (Figure 9(c)). GSEA was performed to find possible biological functions of high CBX8 protein expression of these three tumors, as follows: the cell cycle, DNA replication, linoleic acid metabolism, Wnt signaling pathway, and other tumor signaling pathways (Figure 10).

## 4. Discussion

Cancer is a main public health issue and a major reason for death globally. In 2021, 1,898,160 new cancer cases and 608,570 cancer deaths occurred in the U.S. [14]. Global cancer statistics show that liver cancer is the fifth most typical cancer and the third major reason for global cancer-associated mortality [15]. The recurrence rate is up to 70% even after traditional treatments, like radiofrequency ablation, arterial embolization, chemotherapy, and surgery [16]. KIRC takes up 80% of all renal cancers and features poor prognoses [17]. The global annual mortality cases are approximately 90,000, and 25–30% of patients have metastasis at initial diagnosis [18]. OV is the most lethal gynecologic malignancy, with over 125,000 women dying each year globally. The high mortality results from its advanced stage when OV patients are diagnosed and lacking available oriented therapies [19]. Endeavors at earlier test and new therapeutic methods for reducing mortality of LIHC, KIRC, and OV have been greatly unsuccessful since their pathogenesis and origin are poorly understood. CBX8, as an oncogene, plays a role in developing these cancers we mentioned.

Many studies have implied that CBX8 is highly associated with malignant tumor occurrence and development [20–22], but its relationship with LIHC, KIRC, and OV and supporting regulatory mechanisms remains elusive. The study focused on the abnormal CBX8 expression in LIHC, KIRC, and OV, revealing its correlation with clinicopathological features and prognoses.

Higher *CBX8* gene expression was discovered in LIHC, KIRC, and OV tumor tissues than nontumor tissues, following many studies [1, 23, 24]. Subgroup analyses implied that nontumor groups featured a lower *CBX8* expression than those with phase I in LICH and OV, suggesting *CBX8* can be a valid biomarker for earlier diagnoses of LICH and OV (Figure 1). Many studies proved that CBX8 could promote tumor development and metastasis in many cancers, such as breast cancer, hepatocellular carcinoma, cervical cancer cell, and muscle-invasive bladder cancer [23, 25–27]. Our study found that in LIHC patients, *CBX8* expression was obviously higher in stages II-III than in stage I. In KIRC patients, *CBX8* expression was obviously higher in stage IV than in stage I, suggesting that *CBX8* can be a supervision index for distant tumor metastases in LIHC and KIRC. According to the CPTAC database, higher CBX8 protein expression was observed in LIHC, KIRC, and OV tissues than in normal tissues (*p* < 0.05, Figures 1 and 2). Based on the Human Protein Atlas database, we also found higher CBX8 protein expression in LIHC, KIRC, and OV tissues than in normal tissues (Figure 3). Immunohistochemistry outcomes implied that CBX8 was more strongly expressed in tumor tissues than in normal tissues, almost aligning with mRNA and protein expression. These results suggested that CBX8 was upregulated in these three tumors and could be a suitable tumor biomarker.

For LIHC and KIRC patients, some investigations have exhibited that high CBX8 expression induces tumorigenesis and implies poor prognoses [17, 23, 28]. However, no studies focused on the correlation between OV cancer and CBX8. For the first time, our study found that high *CBX8* expression indicated the worst prognosis in OV patients. Further investigation indicated that patients having higher CBX8 expression featured shorter OS, RFS, and DFS in LIHC, KIRC, and OV (expect OS) (Figure 4), implying that CBX8 might constitute a key molecule in the prognosis supervision of patients with these tumors we mentioned. Cox regression analyses suggested that CBX8 constituted an IPF for OS and DFS of LIHC, KIRC, and OV patients, and it constituted an IPF for RFS of KIRC and OV patients. It implies that CBX8 expression includes a bona fide index for recurrence in patients with LIHC, KIRC, and OV, suggesting that it may be a potentially valuable prognostic and diagnostic biomarker for LIHC, KIRC, and OV.

DNA methylation is a typical epigenetic mechanism presented in each form of cancer. Substantial evidence exhibited that gene methylation caused RNA transcription suppression, resulting in upregulating oncogenes or downregulating inhibitor genes and ultimately affecting tumor formation [29]. The link between DNA methylation (cg07581365 site) of CBX8 and the prognoses of LIHC, KIRC, and OV patients was investigated (Figure 6). Using the MethSurv instrument, we identified that KIRC patients with lower CBX8 methylation had a worse survival time than those with higher methylation (*p* < 0.05); LIHC patients had the same trend. *CBX8* methylation could cut *CBX8* expression, thus improving patient prognoses. However, higher CBX8 gene methylation in OV patients can result in a worse survival time. It may be caused by the insertion of methylation sites that can promote oncogenes. Gene mutations are highly associated with tumors and are typically related to poor prognoses. However, CBX8 genetic change percentage in LIHC, KIRC, and OV was approximately 3%, and the genetic change exhibited no obvious relation to a poor OS and RFS (Figure 5).

However, it has also been revealed that CBX8 is associated with tumor-infiltration immune cells and may affect tumor recurrence and progression [30–32]. TMB, MSI, purity, and ploidy are biomarkers monitoring the efficacy of immunotherapeutic response [33]. According to our findings in LIHC, CBX8 was associated with the number of MIS, purity, and ploidy. In KIRC, CBX8 has close interaction with TMB. In OV patients, CBX8 interactions with purity were close, indicating that CBX8 could participate in modulating the immune response in LIHC, KIRC, and OV. Our investigation implied that CBX8 was positively or negatively linked to different immune cells in three cancers, indicating the potential immunotherapy approaches to cure these diseases (Figure 8).

By TCGA database, as for RNA level, we found the up/downregulation of CBX8-related genes in LIHC, KIRC, and OV cancer (Figure S1) and identified the same upregulated genes in these three tumors (Figure S2). These findings can guide further analysis of interaction genes in tumor generation mechanisms of these cancers. Then, the PPI network was developed for protein levels to find CBX8-related proteins. Based on combined scores, three genes, EED, BMI1, and RNF2, were chosen as candidate molecules for cooperating with CBX8 to further assess prognoses in LIHC patients. Fortunately, the outcomes implied that HH groups featured the shortest OS, RFS, and DFS, and LL groups exhibited the longest OS, RFS, and DFS, implying that overexpressions of CBX8, EED, BMI1, and RNF2 were linked to poorer prognoses in LIHC (Figure S3). Previous investigations reported that CBX8 works with EED for DNA damage repair [24]. CBX8, BMI1, and RNF2 are interaction proteins that maintain transcriptional repression of hundreds of cancer growth and signaling-related genes [34]. Therefore, these results suggest that CBX8 and EED, BMI1, and RNF2 feature a concerted effort to promote tumors.

GO analyses uncovered that CBX8 was gathered in the biological process, cell cycle regulation, and molecular functions, which Choi et al. approved [35] (Figure 9(b)). Meanwhile, KEGG outcomes revealed that CBX8 was majorly centered in transcriptional misregulation in cancer, signaling pathways regulating pluripotency of stem cells, systemic lupus, erythematosus, and other processes (Figure 9(c)), consistent with van Wijnen et al.'s report [2]. Yuan et al. found that CBX8 promotes muscle-invasive bladder cancer through the p53 signaling pathway [25]. Chris discovered CBX8 via the AKT/*β*-Catenin signaling pathway to affect liver cancer development [36]. In our study, GSEA was performed to find possible associated biological functions and signaling pathways of these three cancers with higher CBX8 expression. We found CBX8 enrichments in the cell cycle, DNA replications, linoleic acid metabolism, Wnt signaling pathway, and other tumor signaling pathways. These findings could be a significant guide for our later investigation of mechanisms of tumor geneses and progression in LIHC, KIRC, and OV patients (Figure 10).

Overall, CBX8 may be a potential prognostic and diagnostic biomarker for early diagnosis and prognosis monitoring and a therapeutic target for LIHC, KIRC, and OV patients. The diagnostic and prognostic values of CBX8 should be further explored by in vivo and in vitro studies. Further mechanistic studies are required to validate our findings and promote the clinical application.

## Figures and Tables

**Table 1:** Correlation between *CBX8* expression and clinicopathological characteristics of LIHC.

Characteristics	*CBX8* expression		*χ* ^2^	*p*
	Low (*n* = 193)	High (*n* = 178)		
Gender				
Male	129 (34.77%)	121 (32.61%)	0.015076	0.9023
Female	64 (17.25%)	57 (15.36%)		
Age, years				
<60	89 (24.05%)	80 (21.62%)	0.005224	0.9424
≥60	104 (28.11%)	97 (26.22%)		
TNM stage				
I	101 (29.11%)	70 (20.17%)	7.0821	0.0602
II	38 (10.95%)	48 (13.83%)		
III	39 (11.24%)	46 (13.26%)		
IV	2 (0.58%)	3 (0.86%)		
Grade				
G1	36 (9.84%)	19 (5.19%)	10.249	0.0163^∗^
G2	96 (26.23%)	81 (22.13%)		
G3	52 (14.21%)	70 (19.13%)		
G4	4 (1.09%)	8 (2.19%)		

^∗^ *p* < 0.05 was considered statistically significant.

**Table 2:** Correlation between *CBX8* expression and clinicopathological characteristics of KIRC.

Characteristics	*CBX8* expression			
	Low (*n* = 265)	High (*n* = 265)	*χ* ^2^	*p*
Gender				
Male	167 (31.51%)	177 (33.40%)	0.67095	0.4127
Female	98 (18.49%)	88 (16.60%)		
Age, years				
<60	123 (23.21%)	122 (23.02%)	0	1
≥60	142 (26.79%)	143 (26.98%)		
TNM stage				
I	137 (26.00%)	128 (24.29%)	2.1651	0.5388
II	31 (5.88%)	26 (4.93%)		
III	59 (11.20%)	64 (12.14%)		
IV	36 (6.83%)	46 (8.73%)		
Grade				
G1	7 (1.34%)	7 (1.34%)	8.9854	0.02949^∗^
G2	124 (23.75%)	103 (19.73%)		
G3	102 (19.54%)	104 (19.92%)		
G4	26 (4.98%)	49 (9.39%)		

^∗^ *p* < 0.05 was considered statistically significant.

**Table 3:** Correlation between *CBX8* expression and clinicopathological characteristics of OV Patients.

Characteristics	*CBX8* expression			
	Low (*n* = 190)	High (*n* = 189)	*χ* ^2^	*p*
Age, years				
<60	107 (28.23%)	92 (24.27%)	1.9212	0.166
≥60	83 (21.90%)	97 (25.59%)		
TNM stage				
I-II	8 (2.13%)	16 (4.26%)	10.976	0.004^∗^
III	141 (37.50%)	154 (40.96%)		
IV	39 (10.37%)	18 (4.79%)		
Grade				
G1-G2	18 (4.88%)	28 (7.59%)	1.6411	0.200
G3-G4	163 (44.17%)	160 (43.36%)		

^∗^ *p* < 0.05 was considered statistically significant.

**Table 4:** Univariate and multivariate analyses of different parameters for overall survival, relapse-free survival, and disease-free survival in LIHC patients.

Carcinoma	Survival	Characteristics	Univariate analysis			Multivariate analysis		
			*p*	HR	95% CI	*p*	HR	95% CI
LIHC	OS	*CBX8* expression high vs. low	<0.001^∗^	2.6	1.7-3.8	<0.001^∗^	2.5	1.7-3.9
		Gender male vs. female	0.26	0.81	0.57-1.2			
		Age, years ≥60 vs. <60	0.27	1.2	0.86-1.7			
		TNM stage III/IV vs. I/II	<0.001^∗^	2.4	1.7-3.5	<0.001^∗^	2.4	1.7-3.5
		Grade G3/G4 vs. G1/G2	0.54	1.1	0.78-1.6			
	RFS	*CBX8* expression high vs. low	0.028^∗^	1.4	1-1.9	0.086	1.3	0.96-1.8
		Gender male vs. female	0.94	0.99	0.72-1.4			
		Age, years ≥60 vs. <60	0.75	1	0.78-1.4			
		TNM stage III/IV vs. I/II	<0.001^∗^	2.4	1.7-3.3	<0.001^∗^	2.3	1.7-3.2
		Grade G3/G4 vs. G1/G2	0.52	1.1	0.81-1.5			
	DFS	*CBX8* expression high vs. low	<0.001^∗^	1.9	1.4-2.6	<0.001^∗^	1.9	1.4-2.6
		Gender male vs. female	0.42	0.89	0.68-1.2			
		Age, years ≥60 vs. <60	0.37	1.1	0.86-1.5			
		TNM stage III/IV vs. I/II	<0.001^∗^	2.1	1.6-2.8	<0.001^∗^	2.1	1.6-2.8
		Grade G3/G4 vs. G1/G2	0.63	1.1	0.81-1.4			

**Table 5:** Univariate and multivariate analyses of different parameters for overall survival, relapse-free survival, and disease-free survival in KIRC patients.

Carcinoma	Survival	Characteristics	Univariate analysis			Multivariate analysis		
			*p*	HR	95% CI	*p*	HR	95% CI
KIRC	OS	*CBX8* expression high vs. low	<0.001^∗^	2.5	1.9-3.5	<0.001^∗^	2.2	1.6-3
		Gender male vs. female	0.68	0.93	0.68-1.3			
		Age, years ≥60 vs. <60	<0.001^∗^	1.8	1.3-2.5	0.063	1.4	0.98-1.9
		TNM stage III/IV vs. I/II	<0.001^∗^	4.3	3.1-5.9	<0.001^∗^	3.2	2.2-4.5
		Grade G3/G4 vs. G1/G2	<0.001^∗^	2.6	1.8-3.7	0.005^∗^	1.7	1.2-2.5
	RFS	*CBX8* expression high vs. low	<0.001^∗^	2.4	1.6-3.7	0.010^∗^	1.7	1.1-2.6
		Gender male vs. female	0.058	1.5	0.99-2.3	0.093	1.4	0.94-2.2
		Age, years ≥60 vs. <60	0.27	1.2	0.85-1.8			
		TNM stage III/IV vs. I/II	<0.001^∗^	6	3.9-9	<0.001^∗^	4.7	3-7.2
		Grade G3/G4 vs. G1/G2	<0.001^∗^	3.5	2.2-5.4	0.001^∗^	2.1	1.3-3.4
	DFS	*CBX8* expression high vs. low	<0.001^∗^	2.3	1.7-3	<0.001^∗^	1.9	1.4-2.5
		Gender male vs. female	0.39	1.1	0.85-1.5			
		Age, years ≥60 vs. <60	<0.001^∗^	1.7	1.3-2.3	0.036^∗^	1.4	1-1.8
		TNM stage III/IV vs. I/II	<0.001^∗^	4.4	3.3-5.9	<0.001^∗^	3.4	2.5-4.6
		Grade G3/G4 vs. G1/G2	<0.001^∗^	2.8	2-3.8	<0.001^∗^	1.9	1.3-2.6

**Table 6:** Univariate and multivariate analyses of different parameters for overall survival, relapse-free survival and disease-free survival in OV patients.

Carcinoma	Survival	Characteristics	Univariate analysis			Multivariate analysis		
			*p*	HR	95% CI	*p*	HR	95% CI
OV	RFS	*CBX8* expression high vs. low	0.001^∗^	1.8	1.3-2.6	0.002^∗^	1.8	1.2-2.5
		Gender male vs. female	0.98	1	0.78-1.3			
		Age, years ≥60 vs. <60	0.17	1.5	0.85-2.5	0.21	1.4	0.82-2.4
		TNM stage III/IV vs. I/II	0.34	1.2	0.83-1.7			
		Grade G3/G4 vs. G1/G2	0.001^∗^	1.8	1.3-2.6	0.002^∗^	1.8	1.2-2.5
	DFS	*CBX8* expression high vs. low	0.012^∗^	1.6	1.1-2.2	0.009^∗^	1.8	1.3-2.6
		Gender male vs. female	0.31	1.1	0.9-1.4			
		Age, years ≥60 vs. <60	0.05	1.7	1-2.9	0.13	1.5	0.88-2.7
		TNM stage III/IV vs. I/II	0.18	1.3	0.9-1.8	0.25	1.2	0.86-1.8
		Grade G3/G4 vs. G1/G2	0.012^∗^	1.6	1.1-2.2	0.009^∗^	1.8	1.3-2.6

## Data Availability

The data included in the current study were available in TCGA database (https://cancergenome.nih.gov/), GTEx database (https://commonfund.nih.gov/GTEx), CPTAC database (https://cptac-data-portal.georgetown.edu/datasets), and Human Protein Atlas database (https://www.protectatlas.org).

## Supplementary Materials

Supplementary Materials Figure S1: analysis of CBX8-related upregulation and downregulation genes in higher CBX8 samples across LIHC, KIRC, and OV. Figure S2: analysis of the same CBX8-related upregulation genes in LIHC, KIRC, and OV.3. Figure S3: the survival outcomes in liver hepatocellular carcinoma patients with different gene combinations (CBX8-EED; CBX8- BMI1, and CBX8-RNF2). (A), (B), and (C) Pearson's correlation analysis between CBX8 and EED, BMI1, and RNF2, respectively; (D), (E), and (F) OS for different gene combinations; (G), (H), and (I) RFS for different gene combinations. (J), (K), and (L) DFS for different gene combinations. Colored images are available online. Table S1: the upregulated and downregulated CBX8-relevant genes ranked by ∣log2fold change∣ in LIHC. Table S2: the upregulated and downregulated CBX8-relevant genes ranked by ∣log2fold change∣ in KIRC. Table S3: the upregulated and downregulated CBX8-relevant genes ranked by ∣log2fold change∣ in OV. Table S4: the 40 same upregulation genes in LIHC, KIRC, and OV.
