# Supplementary material for: Potential Diagnostic and Prognostic Values of CBX8 Expression in Liver Hepatocellular Carcinoma, Kidney Renal Clear Cell Carcinoma, and Ovarian Cancer: A Study Based on TCGA Data Mining
Source: Comput Math Methods Med. 2022 Jun 29;2022:1372879. doi: 10.1155/2022/1372879 (PMC9259361; doi:10.1155/2022/1372879)
Supplement: Supplementary Materials — Figure S1: analysis of CBX8-related upregulation and downregulation genes in higher CBX8 samples across LIHC, KIRC, and OV. Figure S2: analysis of the same CBX8-related upregulation genes in LIHC, KIRC, and OV.3. Figure S3: the survival outcomes in liver hepatocellular carcinoma patients with different gene combinations (CBX8-EED; CBX8- BMI1, and CBX8-RNF2). (A), (B), and (C) Pearson's correlation analysis between CBX8 and EED, BMI1, and RNF2, respectively; (D), (E), and (F) OS for different gene combinations; (G), (H), and (I) RFS for different gene combinations. (J), (K), and (L) DFS for different gene combinations. Colored images are available online. Table S1: the upregulated and downregulated CBX8-relevant genes ranked by ∣log2fold change∣ in LIHC. Table S2: the upregulated and downregulated CBX8-relevant genes ranked by ∣log2fold change∣ in KIRC. Table S3: the upregulated and downregulated CBX8-relevant genes ranked by ∣log2fold change∣ in OV. Table S4: the 40 same upregulation genes in LIHC, KIRC, and OV. [file 1372879.f1.zip › tableS1-S4.docx]

**Table S1** The upregulated and downregulated CBX8-relevant genes ranked by |log2fold change| in LIHC

| upregulated *CBX8*-relevant genes | | | | | downregulated *CBX8*-relevant genes | | | | |
| --- | --- | --- | --- | --- | --- | --- | --- | --- | --- |
| Gene | baseMean | log2fold change | lfcSE | *P* value | Gene | baseMean | log2fold change | lfcSE | *P* value |
| LGALS14 | 20.65676 | 5.214001 | 0.71466 | 2.97E-13 | CLEC4M | 27.82644 | 3.89293 | 0.33996 | 2.32E-30 |
| CEACAM7 | 49.83672 | 4.749949 | 0.58144 | 3.10E-16 | CLEC1B | 28.49626 | 3.64288 | 0.29988 | 5.89E-34 |
| BRDT | 6.71328 | 4.347446 | 0.41013 | 2.97E-26 | HAMP | 2889.246 | 3.27741 | 0.27435 | 6.80E-33 |
| TSPY2 | 2.923427 | 4.314661 | 0.77030 | 2.13E-08 | CLEC4G | 91.94452 | 3.19693 | 0.24803 | 5.18E-38 |
| NTS | 1709.476 | 4.220664 | 0.38456 | 5.02E-28 | GDF2 | 28.75349 | 3.12685 | 0.46848 | 2.48E-11 |
| SEPTIN14 | 8.573602 | 4.010358 | 0.46064 | 3.14E-18 | BMP10 | 1.622257 | 3.06862 | 0.52785 | 6.12E-09 |
| AGR2 | 631.6722 | 3.704856 | 0.36043 | 8.76E-25 | SAA2 | 19814.74 | 2.97233 | 0.27884 | 1.57E-26 |
| SLITRK1 | 4.061583 | 3.616138 | 0.50546 | 8.42E-13 | RNA5SP334 | 1.357677 | 2.74455 | 0.39378 | 3.17E-12 |
| OLIG3 | 1.712521 | 3.561211 | 0.71965 | 7.48E-07 | ADIPOQ | 1.596644 | 2.71345 | 0.48055 | 1.64E-08 |
| PLAC4 | 6.862968 | 3.547295 | 0.35345 | 1.06E-23 | ANGPTL7 | 9.643592 | 2.68829 | 0.43704 | 7.69E-10 |
| WNT7B | 64.09729 | 3.366501 | 0.31314 | 5.87E-27 | FAM83A-AS1 | 492.1599 | 2.54224 | 0.33090 | 1.56E-14 |
| SPATA31D1 | 3.355459 | 3.324576 | 0.66769 | 6.38E-07 | SAA2-SAA4 | 1985.175 | 2.47282 | 0.27358 | 1.59E-19 |
| FTHL17 | 1.703437 | 3.320924 | 0.69606 | 1.83E-06 | PNMA6F | 1.744797 | 2.44328 | 0.45630 | 8.58E-08 |
| EEF1DP5 | 4.190574 | 3.318933 | 0.42466 | 5.47E-15 | SAA1 | 55593.86 | 2.43165 | 0.27036 | 2.38E-19 |
| NKX6-3 | 2.177424 | 3.290877 | 0.54669 | 1.75E-09 | OR1N2 | 1.066772 | 2.30167 | 1.07093 | 0.031616 |
| CA9 | 638.4736 | 3.270956 | 0.31036 | 5.69E-26 | CRHBP | 190.8284 | 2.27622 | 0.21322 | 1.33E-26 |
| LOC729141 | 1.309754 | 3.26494 | 0.71516 | 4.99E-06 | UBTFL10 | 1.125747 | 2.22147 | 0.33698 | 4.33E-11 |
| FA2H | 80.80177 | 3.251477 | 0.28497 | 3.73E-30 | MT1JP | 4.644086 | 2.21155 | 0.33683 | 5.18E-11 |
| SIX3 | 2.953114 | 3.234835 | 0.40732 | 1.99E-15 | HBD | 3.483309 | 2.12489 | 0.32064 | 3.42E-11 |
| MED15P9 | 2.829785 | 3.188076 | 0.51208 | 4.79E-10 | NDST3 | 3.922588 | 2.10932 | 0.24799 | 1.80E-17 |

**Table S2** The upregulated and downregulated *CBX8*-relevant genes ranked by |log2fold change| in KIRC

| upregulated CBX8-relevant genes | | | | | downregulated CBX8-relevant genes | | | | |
| --- | --- | --- | --- | --- | --- | --- | --- | --- | --- |
| Gene | baseMean | log2fold change | lfcSE | *P* value | Gene | baseMean | log2fold change | lfcSE | *P* value |
| MAGEB2 | 1.943274 | 3.03027 | 0.46936 | 1.07E-10 | AQP6 | 305.9576 | 4.49623 | 0.25481 | 1.10E-69 |
| SSX1 | 3.689136 | 3.01937 | 0.38640 | 5.54E-15 | RHCG | 618.8422 | 4.45968 | 0.27042 | 4.21E-61 |
| RTL1 | 10.16421 | 2.85651 | 0.37791 | 4.07E-14 | FOXI1 | 80.80358 | 3.95714 | 0.34899 | 8.43E-30 |
| PASD1 | 1.247476 | 2.77529 | 0.49243 | 1.74E-08 | BSND | 40.88736 | 3.61743 | 0.35019 | 5.16E-25 |
| ACTBP12 | 8.984348 | 2.77118 | 0.26364 | 7.68E-26 | HEPACAM2 | 79.15819 | 3.58431 | 0.28681 | 7.73E-36 |
| RNY3 | 0.753196 | 2.67687 | 0.91618 | 0.00348 | ATP6V1G3 | 61.84976 | 3.4453 | 0.52694 | 6.22E-11 |
| PAGE2 | 1.82286 | 2.64916 | 0.28355 | 9.38E-21 | TMEM213 | 420.9171 | 3.2945 | 0.26304 | 5.45E-36 |
| HP | 1681.812 | 2.64172 | 0.26251 | 8.04E-24 | TMPRSS11E | 17.88925 | 3.15735 | 0.31669 | 2.06E-23 |
| GOLGA6L7 | 21.03951 | 2.63072 | 0.22521 | 1.59E-31 | GPRC6A | 5.980429 | 2.94128 | 0.48774 | 1.64E-09 |
| HPR | 26.55304 | 2.61737 | 0.25787 | 3.32E-24 | SFTPB | 59.13018 | 2.86404 | 0.23012 | 1.47E-35 |
| LOC101928516 | 5.050447 | 2.54920 | 0.33540 | 2.95E-14 | PLA2G4F | 66.38273 | 2.70653 | 0.27046 | 1.42E-23 |
| APOC3 | 48.51625 | 2.54594 | 0.28967 | 1.51E-18 | DMRT2 | 50.6182 | 2.68095 | 0.26414 | 3.32E-24 |
| IGFN1 | 198.1189 | 2.52349 | 0.24917 | 4.17E-24 | UMOD | 357.7919 | 2.61111 | 0.29517 | 9.06E-19 |
| KRT20 | 9.540374 | 2.45994 | 0.24099 | 1.83E-24 | CLCNKB | 322.8501 | 2.35934 | 0.20646 | 3.04E-30 |
| TNNI3 | 11.81691 | 2.38176 | 0.26879 | 7.93E-19 | GCGR | 39.90682 | 2.28771 | 0.28871 | 2.30E-15 |
| LBP | 2146.352 | 2.28210 | 0.26636 | 1.06E-17 | LRRTM1 | 7.89408 | 2.2282 | 0.35345 | 2.90E-10 |
| RMRP | 2.814204 | 2.25800 | 0.36519 | 6.28E-10 | SLC24A2 | 15.50897 | 2.12792 | 0.24545 | 4.34E-18 |
| S100A7 | 3.146733 | 2.24791 | 0.26870 | 5.97E-17 | ATP6V0A4 | 386.7141 | 2.11689 | 0.26328 | 8.96E-16 |
| LINC00261 | 4.325247 | 2.17249 | 0.25670 | 2.61E-17 | AQP2 | 112.62 | 2.02495 | 0.31534 | 1.35E-10 |
| ANXA8 | 9.874888 | 2.16116 | 0.25380 | 1.66E-17 | ATP6V1B1 | 260.2491 | 2.01533 | 0.18457 | 9.36E-28 |

**Table S3** The upregulated and downregulated CBX8-relevant genes ranked by |log2fold change| in OV

| upregulated CBX8-relevant genes | | | | | downregulated CBX8-relevant genes | | | | |
| --- | --- | --- | --- | --- | --- | --- | --- | --- | --- |
| Gene | baseMean | log2fold change | lfcSE | *P* value | Gene | baseMean | log2fold change | lfcSE | *P* value |
| C2orf69P1 | 1.937562 | 4.088535 | 0.97252 | 2.62E-05 | APCS | 1.613054 | 2.38825 | 0.60012 | 6.90E-05 |
| SEPTIN7P11 | 4.363676 | 3.958312 | 0.42038 | 4.68E-21 | LOC100505797 | 0.94638 | 2.25927 | 0.42436 | 1.02E-07 |
| LINC01608 | 3.326473 | 3.361314 | 0.62873 | 8.98E-08 | GLYATL2 | 148.3733 | 1.78041 | 0.30727 | 6.86E-09 |
| ZNF317P1 | 3.498974 | 3.281926 | 0.41882 | 4.65E-15 | SPATA8 | 0.866259 | 1.67786 | 0.40407 | 3.29E-05 |
| PAGE2 | 94.35165 | 3.223793 | 0.43034 | 6.82E-14 | LYPD2 | 1682.008 | 1.64906 | 0.27364 | 1.68E-09 |
| ACTRT2 | 1.260567 | 3.000194 | 0.55897 | 7.99E-08 | LYNX1 | 2670.407 | 1.6368 | 0.19103 | 1.05E-17 |
| LINC00237 | 14.20307 | 2.941521 | 0.44392 | 3.44E-11 | ZBTB16 | 196.2214 | 1.56033 | 0.19115 | 3.28E-16 |
| NPFFR2 | 20.47431 | 2.91705 | 0.26642 | 6.70E-28 | KRT6A | 1568.262 | 1.55823 | 0.20077 | 8.40E-15 |
| NAA11 | 7.669051 | 2.824581 | 0.46787 | 1.57E-09 | C6orf15 | 288.8959 | 1.46086 | 0.25662 | 1.25E-08 |
| GAGE2B | 20.50774 | 2.721705 | 0.62020 | 1.14E-05 | MYL1 | 0.653474 | 1.44932 | 0.51769 | 0.005117 |
| GAGE2A | 20.50774 | 2.721705 | 0.62020 | 1.14E-05 | LNCAROD | 1.329386 | 1.44129 | 0.25714 | 2.08E-08 |
| GAGE8 | 20.50774 | 2.721705 | 0.62020 | 1.14E-05 | LOC105378305 | 1.329386 | 1.44129 | 0.25714 | 2.08E-08 |
| CT83 | 1.290209 | 2.688457 | 0.85705 | 0.001708 | SLCO1B3 | 26.53958 | 1.4302 | 0.27682 | 2.38E-07 |
| PAGE2B | 12.21429 | 2.67418 | 0.30494 | 1.79E-18 | SAA4 | 12.98222 | 1.42047 | 0.25209 | 1.75E-08 |
| LINC01580 | 2.415707 | 2.651545 | 0.60458 | 1.16E-05 | FOXI1 | 48.86515 | 1.39305 | 0.25359 | 3.95E-08 |
| MYF5 | 1.204221 | 2.60052 | 0.59609 | 1.29E-05 | CEACAM5 | 30.46254 | 1.39243 | 0.32033 | 1.38E-05 |
| GFY | 11.89263 | 2.580995 | 0.23726 | 1.47E-27 | IGFL2-AS1 | 120.0463 | 1.37917 | 0.25377 | 5.49E-08 |
| PTF1A | 13.02034 | 2.544476 | 0.37504 | 1.16E-11 | EPYC | 848.085 | 1.34681 | 0.26953 | 5.83E-07 |
| SLC25A24P1 | 5.225791 | 2.504295 | 0.37406 | 2.16E-11 | ADH1B | 437.8021 | 1.3395 | 0.27105 | 7.74E-07 |
| NRG1-IT1 | 1.073541 | 2.50293 | 0.63140 | 7.37E-05 | PTGER2 | 536.6948 | 1.33723 | 0.15063 | 6.82E-19 |

**Table S4** the 40 same upregulation genes in LIHC, KIRC and OV

| Genes | | | | |
| --- | --- | --- | --- | --- |
| CASC20 | FOXJ1 | LOC100128674 | MAGEC1 | PAGE2 |
| FAM230C | PRAC2 | RBPJL | SRGAP3-AS2 | CDHR4 |
| TAGLN3 | VCX3A | DCAF4L2 | GAS2L2 | RFX6 |
| MED15P9 | MIR663AHG | NAA11 | MAGEB2 | MAGEC3 |
| LINC00355 | TLX2 | LINC02616 | NR0B1 | LHX5 |
| PAGE2B | ZSCAN10 | DPYSL5 | LINC01667 | PASD1 |
| DCAF8L2 | ACTL8 | LINC01518 | LOC349160 | LINC02511 |
| LINC02055 | LINC00668 | LINC01446 | MAGEB1 | MCIDAS |
