# Supplementary figures and images for: Potential Diagnostic and Prognostic Values of CBX8 Expression in Liver Hepatocellular Carcinoma, Kidney Renal Clear Cell Carcinoma, and Ovarian Cancer: A Study Based on TCGA Data Mining
Source: Comput Math Methods Med. 2022 Jun 29;2022:1372879. doi: 10.1155/2022/1372879 (PMC9259361; doi:10.1155/2022/1372879)

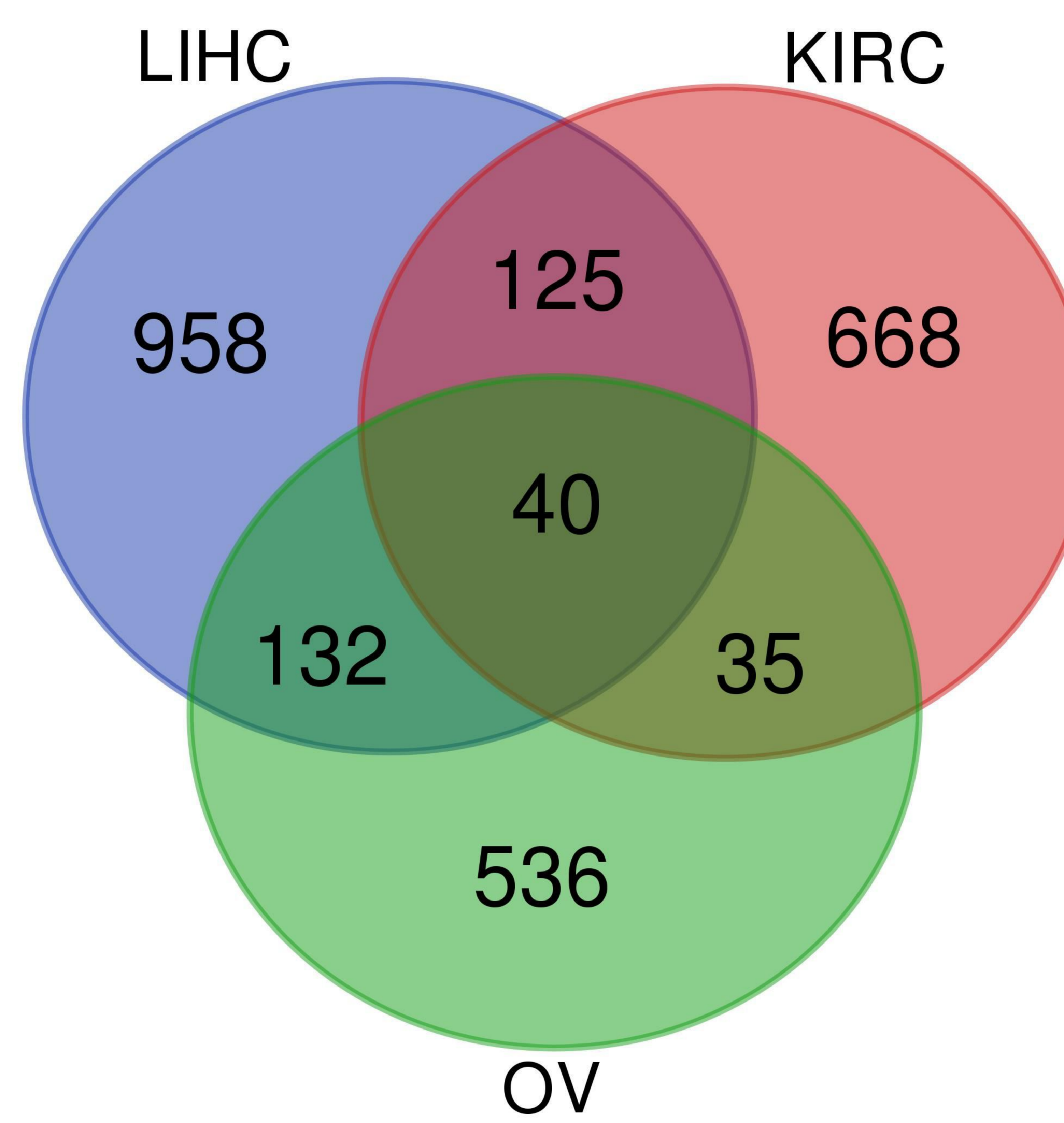

**Figure S2** Analysis of the same CBX8-related upregulation genes in LIHC, KIRC and OV.

Supplement: Supplementary Materials — Figure S1: analysis of CBX8-related upregulation and downregulation genes in higher CBX8 samples across LIHC, KIRC, and OV. Figure S2: analysis of the same CBX8-related upregulation genes in LIHC, KIRC, and OV.3. Figure S3: the survival outcomes in liver hepatocellular carcinoma patients with different gene combinations (CBX8-EED; CBX8- BMI1, and CBX8-RNF2). (A), (B), and (C) Pearson's correlation analysis between CBX8 and EED, BMI1, and RNF2, respectively; (D), (E), and (F) OS for different gene combinations; (G), (H), and (I) RFS for different gene combinations. (J), (K), and (L) DFS for different gene combinations. Colored images are available online. Table S1: the upregulated and downregulated CBX8-relevant genes ranked by ∣log2fold change∣ in LIHC. Table S2: the upregulated and downregulated CBX8-relevant genes ranked by ∣log2fold change∣ in KIRC. Table S3: the upregulated and downregulated CBX8-relevant genes ranked by ∣log2fold change∣ in OV. Table S4: the 40 same upregulation genes in LIHC, KIRC, and OV. [file 1372879.f1.zip › FigureS2.pdf]
